# Small Dense Low Density Lipoprotein Particles Are Associated with Poor Outcome after Angioplasty in Peripheral Artery Disease

**DOI:** 10.1371/journal.pone.0108813

**Published:** 2014-09-29

**Authors:** Vincenzo Jacomella, Philipp A. Gerber, Kathrin Mosimann, Marc Husmann, Christoph Thalhammer, Ian Wilkinson, Kaspar Berneis, Beatrice R. Amann-Vesti

**Affiliations:** 1 Clinic for Angiology, University Hospital Zurich, Zurich, Switzerland; 2 Clinic for Endocrinology, University Hospital Zurich, Zurich, Switzerland; 3 Clinical Pharmacology Unit, University of Cambridge, Cambridge, United Kingdom; Los Angeles, United States of America

## Abstract

**Purpose:**

In patients suffering from symptomatic peripheral artery disease (PAD), percutaneous revascularization is the treatment of choice. However, restenosis may occur in 10 to 60% in the first year depending on a variety of factors. Small dense low density lipoprotein (sdLDL) particles are associated with an increased risk for cardiovascular events, but their role in the process of restenosis is not known. We conducted a prospective study to analyze the association of sdLDL particles with the outcome of balloon angioplasty in PAD. The composite primary endpoint was defined as improved walking distance and absence of restenosis.

**Methods:**

Patients with angiographically documented PAD of the lower extremities who were scheduled for lower limb revascularization were consecutively recruited for the study. At baseline and at three month follow-up triglyceride, total cholesterol, LDL size and subclasses and HDL cholesterol and ankle-brachial index (ABI) were measured. Three months after the intervention duplex sonography was performed to detect restenosis.

**Results:**

Sixty-four patients (53% male) with a mean age of 68.6±9.9 years were included. The proportion of small- dense LDL particles (class III and IV) was significantly lower (33.1±11.0% vs. 39.4±12.1%, p = 0.038) in patients who reached the primary end-point compared with those who did not. Patients with improved walking distance and without restenosis had a significantly higher LDL size at baseline (26.6±1.1 nm vs. 26.1±1.1 nm, p = 0.046) and at follow-up (26.7±1.1 nm vs. 26.2±0.9 nm, p = 0.044) than patients without improvement.

**Conclusions:**

Small-dense LDL particles are associated with worse early outcome in patients undergoing percutaneous revascularization for symptomatic PAD.

## Introduction

Peripheral artery disease (PAD) has a prevalence of up to 20% in the elderly population [1]. The majority of patients are asymptomatic [2]; therefore, early modification of risk factors is mandatory to reduce the high rate of morbidity and mortality associated with PAD [3].

Up to 10–35% of PAD patients are symptomatic [2], with reduced quality of life due to pain and impaired mobility. The presence of chronic wounds and critical ischemia may compromise limb viability. Revascularization with angioplasty is an approved therapeutic option to improve quality of life in patients with intermittent claudication, and the treatment of choice in critical limb ischemia. However, despite new devices and techniques, restenosis is still a major problem, occurring in 10 to 60% of cases after an initially technically successful angioplasty. The rate of restenosis depends on a variety of factors, such as severity of the PAD (i.e. claudication versus critical limb ischemia), the lesion type (occlusion versus stenosis), the quality of both run-in and run-off vessels, the length of the lesions but also on cardiovascular risk factors [2], [4], [5], such as diabetes, hyperlipidemia, hypertension and smoking.

Earlier publications have outlined the importance of low density lipoprotein (LDL) size as a predictor of cardiovascular events and progression of coronary artery disease [6]. The presence of small, dense LDL (sdLDL) particles is an established cardiovascular risk factor by the national Cholesterol Education Program Adult treatment Panel III. sdLDL particle size is a predictive marker for cardiovascular mortality in PAD patients [7], but its role in the process of restenosis and clinical outcome in patients undergoing percutaneous revascularization is unclear. Therefore, we conducted a prospective study to investigate the potential role of sdLDL particle as a predictor of early restenosis and adverse clinical outcome after angioplasty with or without stenting.

## Methods

### Study design and patients

In this prospective, single-center, observational study, the effect of sdLDL particles on restenosis and clinical outcome after endovascular lower limb revascularization in PAD patients was investigated.

Patients with atherosclerotic PAD, Fontaine I–III of the lower limb, with or without a history of peripheral vascular intervention or vascular surgery, who were scheduled for an intervention, were consecutively recruited. Exclusion criteria were cardiac arrhythmia, chronic inflammatory vascular disorders or failed revascularization, defined as a more than 50% residual stenosis confirmed angiographically or by duplex ultrasound after the procedure. All examinations were performed at the study center (Clinic for Angiology, University Hospital Zurich).

After baseline investigation patients underwent peripheral angioplasty with plain balloon angioplasty, with or without stenting (without drug-coated balloon or stent). The decision to implant a stent was left to the operator, but patients receiving a drug-eluting stent were excluded.

At baseline, body mass index (BMI), total cholesterol, LDL cholesterol, HDL cholesterol, triglycerides, LDL-phenotype and ankle-brachial index (ABI) were recorded. Details of other risk factors and medication were recorded, and walking capacity was evaluated using a walking questionnaire (SF-35). At three months follow-up, LDL-phenotype and ABI were determined. A Duplex ultrasound examination to detect restenosis of the target lesion was performed, and walking capacity was assessed with a questionnaire (SF-35).

The primary endpoint was defined as improved walking distance and absence of restenosis.

The local ethics committee (“Kantonale Ethikkommission Zürich”) approved the study and all patients gave written informed consent.

### Laboratory measurements

Triglycerides, total cholesterol and lipoprotein cholesterol values were measured by enzymatic procedures (Abbott ABA 200 instrument). HDL cholesterol was determined by the dextran sulphate-magnesium precipitation procedure. Low-density lipoprotein cholesterol was calculated with the Friedewald formula [8]. To assess LDL particles size and distribution, non-denaturing polyacrylamide gradient gel electrophoresis (GGE) of plasma was performed at 10–14°C in 2–16% polyacrylamide gradient gels. Gels were subjected to electrophoresis for 24 h at 125 V in tris borate buffer (pH 8.3) as described elsewhere [9]. Gels were fixed and stained for lipids in a solution containing Oil Red O in 60% ethanol at 55°C. Gels were placed on a light source and photographed using a Luminescent Image Analyzer, LAS-3000 of Fujifilm. Migration distance for each absorbance peak was determined and the molecular diameter corresponding to each peak was calculated from a calibration curve generated from the migration distance of size standards of known diameter, which includes carboxylated latex beads (Duke Scientific, Palo Alto, CA), thyroglobulin and apoferritin (HMW Std, Pharmacia, Piscataway, NJ) having molecular diameter of 38.0 nm, 17.0 nm and 12.2 nm, respectively, and lipoprotein calibrators of previously determined particle size. LDL subclass distribution (LDL I (272–285 nm), IIA (265–272 nm), IIB (256–265 nm), IIIA (247–256 nm), IIIB (242–247 nm), IVA (233–242 nm) and IVB (220–233 nm)) as percentage of total LDL was calculated.

### Ankle-brachial index and Duplex ultrasound

Ankle-brachial arterial pressure index was assessed with the patient in the supine position. Systolic ankle blood pressure of the posterior and anterior tibial artery, and the peroneal artery on both legs was obtained by hand-held 6 MHz Doppler probe (Kranzbühler, Logidop 2, Pilger Medical Electronics, Switzerland). ABI was calculated as the ratio of the highest ankle systolic blood pressure divided by the highest brachial systolic blood pressure for each leg. The ABI of the treated leg was taken as the study parameter.

Patency of the revascularized segment was assesed at three months follow-up visit with duplex ultrasound (DUS), primary patency was maintained until restenosis (>50% diameter reduction) defined by a peak systolic velocity (PSV) ratio >2.4 was documented by DUS.

### Revascularization procedure

All patients were treated according to guidelines with thrombocyte antiaggregation and/or anticoagulation, statins, and antihypertensive therapy if indicated.

In addition to best medical treatment, patients were treated with balloon angioplasty, stent implantation was additionally performed at the discretion of the interventionalist. Medication remained unchanged except for clopidogrel given for a period of 28 days in case of stent implantation.

### Statistical analysis

Data are presented as means ± SD, or values and percentages. For the analysis of categorical data, the χ^2^ and Fisher's exact test were applied. For comparison of continuous variables in two independent groups, the Mann–Whitney U test was used. A generalized linear model was used for the testing of correlations. A value of p<0.05 was considered significant.

## Results

### Patient characteristics

A total of 64 patients were included in the study (53.1% males, mean age 68.6±9.9 years). Baseline data are summarized in Table 1. Mean ABI was 0.71±0.20. Antiplatelet/anticoagulation medication at baseline included acetylsalicylic acid (90.6%), clopidogrel (53.1%), low molecular weight heparin (14.1%) and oral anticoagulation with phenprocoumon (6.3%). Lipid-lowering agents (statins) were used in 90.6% of patients. Antihypertensive medication included diuretics (37.5%), beta-blocker (32.8%), calcium channel blockers (29.7%), ACE inhibitors (31.3%), angiotensin II receptor antagonists (26.6%) and aldosterone receptor antagonists (6.3%).

**Table 1:** Baseline characteristics of study population (n = 64).

Characteristic	Mean/proportion
Age (years)	69±10
Sex male/female (%)	53/47
BMI (kg/m^2^)	25.2±4.3
Total cholesterol (mmol/l)	4.6±1.1
HDL cholesterol (mmol/l)	1.3±0.6
LDL cholesterol (mmol/l)	2.4±0.9
Triglycerides (mmol/l)	2.1±1.4
Coronary artery disease (%)	28
Cerebrovascular disease (%)	31
Renal insufficiency (%)	22
Arterial Hypertension (%)	83
Diabetes mellitus (%)	30
Smoker	
Current (%)	48
Ever (%)	80

Values are given in absolute numbers (mean±SD) or as percentage.

Most patients were classified as Fontaine stage IIb (50%) and IIa (21%). Only 6% and 1.2% were staged Fontaine stage I or III, respectively. Fotaine stages were defined as follows: Stage I - asymptomatic, incomplete blood vessel obstruction, stage II - mild claudication pain in limb, stage IIA - Claudication at a distance of greater than 200 metres, stage IIB - Claudication distance of less than 200 metres, stage III - rest pain, mostly in the feet, stage IV - necrosis and/or gangrene of the limb.

### Outcome

The combined endpoint of improved walking distance without occurrence of restenosis was reached in 24 (37.5%) patients with a significant improvement of ABI from 0.70±0.20 to 0.93±1.80 (p<0.002). Restenosis or reocclusion of the treated lesion occurred in 11 patients. The use of stents (in 39.1% of patients) did not influence the combined endpoint (reached in 33.3% of patients without stent implantation and in 44% of patients with stent implantation, p = 0.44).

### LDL size

At baseline, mean LDL particle size was 26.3±1.1 nM, and did not differ at follow-up (26.4±1.0 nM, ns). LDL particle size was significantly higher in women than in men at both time points (baseline: 26.6±1.1 nM vs. 26.0±1.1 nM, follow-up: 26.6±0.8 nM vs. 26.1±1.1 nM, p<0.05). There was no correlation between LDL size and age, but there was a negative correlation with BMI at follow-up (p = 0.006), and a trend towards a negative correlation at baseline (p = 0.07).

**Figure 1:**
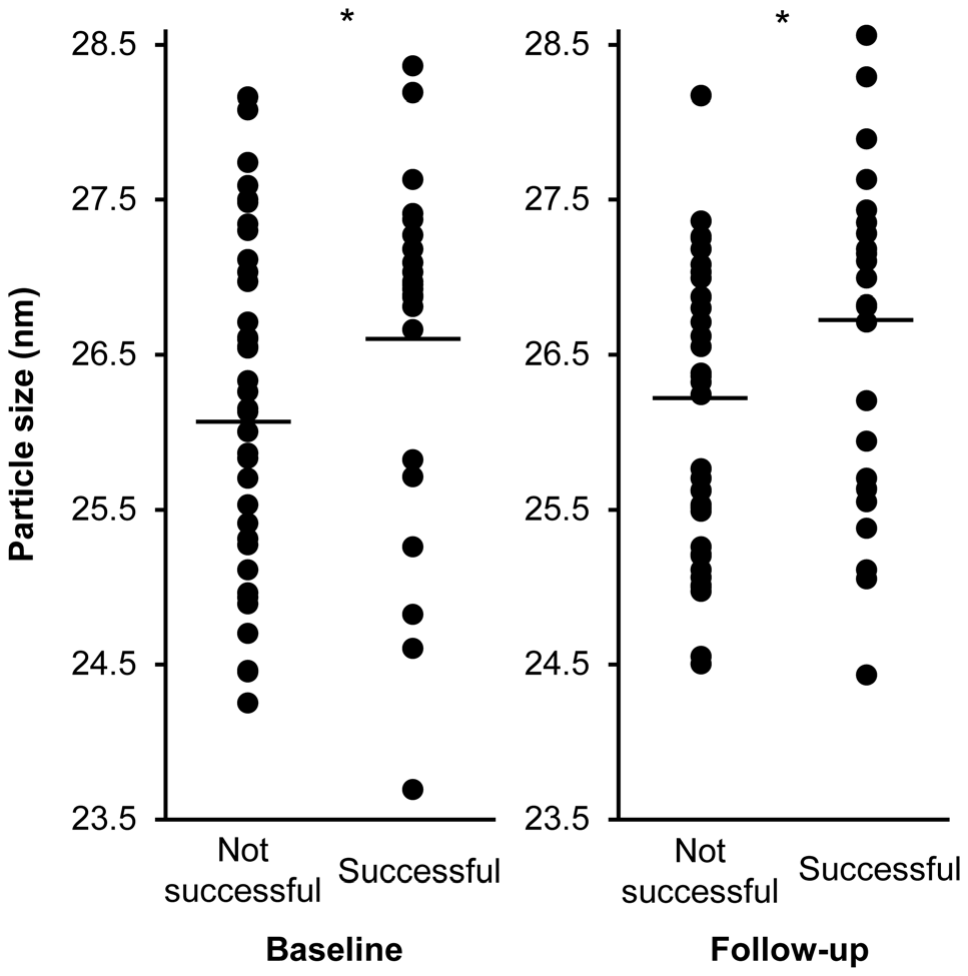
LDL particle size in non-successful (restenosis and/or absence of clinical improvement) vs successful revascularization, * p<0.05.

LDL size at baseline and at follow-up was different between patients achieving the primary end-point and those who did not. Patients with improved walking distance and without restenosis had a significantly higher LDL size at baseline (26.6±1.1 nM vs. 26.1±1.1 nM, p = 0.046) and at follow-up (26.7±1.1 nM vs. 26.2±0.9 nM, p = 0.044, figure 1) than those who did not improve or restenosed.

### LDL particle subclasses

**Figure 2:**
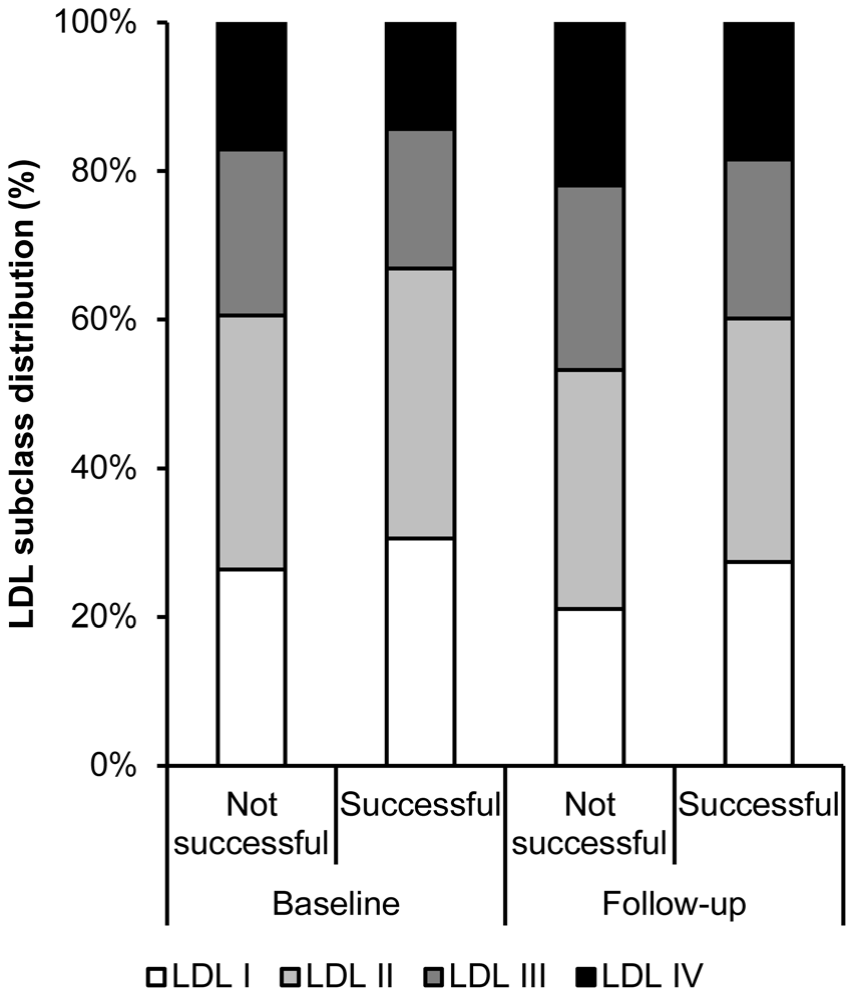
Relative distribution of the different LDL size subclasses (from LDL I = large to LDL IV = small) within the two outcome groups.

The distribution of different LDL subclasses among patients which improvement compared to those without improvement at three months follow-up is shown in figure 2. The proportion of small dense LDL particles (class III and IV) was significantly lower in patients who reached the primary end-point compared to those who did not. These differences were seen at baseline (33.1±11.0% vs. 39.4±12.1%, p = 0.038) and at follow-up (39.8±8.2% vs. 46.8±10.1%, p = 0.008). If the components of the primary endpoint were analyzed separately, a tendency was detected towards a lower proportion of small dense LDL particles in patients without restenosis (compared to those with restenosis) at baseline (36.4±11.6% vs. 40.8±13.2%, p = 0.30) and at follow-up (43.1±10.2% vs. 48.1±8.6%, p = 0.09), and a significant difference was seen in patients with improved walking distance (compared to those without improvement) at baseline (33.6±11.3% vs. 39.5±12%, p = 0.04) and at follow-up (40.9±9.1% vs. 46.4±10%, p = 0.04).

Using a generalized linear model, the effect of different cardiovascular risk factors was assessed. The proportion of small, dense LDL particles at follow-up was still significantly lower in patients who met the end-point after adjustment for gender, smoking status and the diagnoses of arterial hypertension, dyslipidemia or diabetes mellitus (p = 0.03, table 2).

**Table 2:** Multiple linear regression was performed to assess the effect of the amount of small dense LDL particles as well as gender, smoking status and the diagnoses of arterial hypertension, dyslipidemia or diabetes mellitus on the primary outcome.

		95% Confidence Interval		
Parameter	Coefficient	Lower	Upper	p-Value
sdLDL particles (%)	−0.014	−0.026	−0.001	0.03
Female Gender	0.075	−0.156	0.306	0.53
Smoker	0.001	−0.225	0.227	0.99
Hypertension	−0.142	−0.440	0.156	0.35
Dyslipidemia	0.057	−0.189	0.304	0.65
Diabetes	−0.129	−0.380	0.122	0.31

### Other cardiovascular risk factors

In addition to the assessment with a generalized linear model, the individual effect of baseline cardiovascular risk factors – in particular conventional risk factors – other than LDL size was further assessed by analyzing differences between the two outcome groups.

Age was not different in patients who reached the primary end-point compared to those who did not (67.5±10.5 vs. 69.3±9.5, p = 0.69), as was BMI (25.4±4.7 vs. 25.1±4.0 kg/m^2^, p = 0.92) and gender (female gender 58.3% vs. 40.0%, p = 0.20). Further, there was no difference in the concentration of total cholesterol (4.7±1.0 mM vs. 4.5±1.2 mM, p = 0.57), HDL cholesterol (1.4±0.6 mM vs. 1.2±0.5 mM, p = 0.38), LDL cholesterol (2.5±0.6 mM vs. 2.3±1.0 mM, p = 0.07) or triglycerides (1.7±0.9 vs. 2.4±1.7 mM, p = 0.23). Statin therapy was installed in 90.6% of patients at baseline, and started in the remaining 9.4% (6 patients) at the time of admission. The primary outcome did not differ significantly between patients with and without statin therapy (p = 0.19).

## Discussion

The results of this study show that patients with a successful outcome after percutaneous revascularization of PAD have a larger LDL particles size compared to patients who fail to show a clinical improvement or who develop restenosis of the treated vessel. This suggests that small LDL particle size may be used as a predictor of poor outcome after peripheral angioplasty.

A correlation between some lipid markers such as lipoprotein (a) or other serum lipid subfractions and restenosis rate after angioplasty of the peripheral or coronary arteries has already been shown [10]. However, to our knowledge this is the first study investigating the impact of the LDL particle size with respect to restenosis and clinical outcome in claudicants.

Patients with PAD have a high level of small LDL-particles [11], and are more prone to cardiovascular events [6]. In patients with coronary stent implantation, an increase in LDL particle size during follow-up is associated with reduced incidence of in-stent restenosis [12]. Here we show an association with baseline (and follow-up) LDL particle size, but not with its changes during follow-up.

The reasons for the atherogenicity of small LDL particle are varied and not completely understood, it is thought that the small particles are absorbed better by the arterial tissue [13]. In addition, there is probably a greater affinity with the proteoglycans of connective tissue [14] and greater exposure to oxidative processes [15]. Furthermore, a correlation of small dense LDL particles with progressing atherosclerosis has been shown in many studies. Increased intima media thickness is associated with a smaller LDL particle size. In addition to these cross-sectional association studies [16], [17], we collected also prospective data showing a predictive value of the amount of small dense LDL particles at baseline regarding progression of intima media thickness during a follow-up of two years in patients with dysglycemia [18].

Angioplasty causes a mechanical vascular injury with induction of an inflammatory reaction. It is characterized by inflammatory cell infiltration, release of growth factors, medial smooth muscle cell (SMC) modulation and proliferation [19]. Therefore, it is conceivable that all these mechanisms, which lead to neointimal proliferation, are accelerated by the presence of sdLDL particles.

In contrast to sdLDL particles, there was no association of LDL cholesterol levels with the primary endpoint, therefore LDL cholesterol seems not able to predict the outcome of endovascular intervention. This is probably due to the fact that rigorous LDL cholesterol control with statins is installed in patients suffering from PAD. Further, the independence of LDL cholesterol levels and sdLDL particles is underlined by studies describing different effects of certain therapies on LDL cholesterol and sdLDL [20].

The GGE method for determination of LDL particle size and classification has been shown to be reliable, with a high agreement when compared to other methods. However, it should be mentioned that there are also other methods as nuclear magnetic resonance or ion mobility with comparable precision. Further, newer methods have been developed that are convenient to use and may help to establish the use of LDL particle size outside the academic research [21]

The strength of this study is that the data were prospectively assessed in a well-defined cohort of patients with PAD undergoing revascularization. Further, due to the single center design of the study, it was possible that all clinical and biochemical measurements were performed at the same place and by the same investigators, therefore limiting possible inter-observer biases. This was of particular importance with respect to the measurement of LDL size and assessment of restenosis rate. A limitation is the relatively small sample size.

In summary, the presence of high amounts of small dense LDL particles is a negative predictor regarding successful outcome of peripheral angioplasty. Therefore, measurement of this parameter should be considered in patients undergoing balloon angioplasty. Further, therapies targeting LDL particle size and the proportion of sdLDL particles might be considered in patients with high amounts of sdLDL particles in the future to improve clinical outcome after vascular intervention.
